# Good Response of Advanced Thymic Carcinoma with Low PD-L1 Expression to Chemotherapy plus Pembrolizumab as First-Line Therapy and to Pembrolizumab as Maintenance Therapy: A Case Report

**DOI:** 10.3390/ph15070889

**Published:** 2022-07-19

**Authors:** Yoichi Nishii, Kazuki Furuhashi, Kentaro Ito, Tadashi Sakaguchi, Yuta Suzuki, Kentaro Fujiwara, Taro Yasuma, Tetsu Kobayashi, Corina N. D’Alessandro-Gabazza, Esteban C. Gabazza, Osamu Taguchi, Osamu Hataji

**Affiliations:** 1 Respiratory Center, Matsusaka Municipal Hospital, Matsusaka 515-0073, Japan; mchnishii@city-hosp.matsusaka.mie.jp (Y.N.); mchkazuki.f@city-hosp.matsusaka.mie.jp (K.F.); kentarou_i_0214@yahoo.co.jp (K.I.); mchsakaguchi@city-hosp.matsusaka.mie.jp (T.S.); mchyu-ta@city-hosp.matsusaka.mie.jp (Y.S.); mchfujiwara@city-hosp.matsusaka.mie.jp (K.F.); mchosamutaguchi@city-hosp.matsusaka.mie.jp (O.T.); mch1031@city-hosp.matsusaka.mie.jp (O.H.); 2 Department of Immunology, Faculty and Graduate School of Medicine, Mie University, Tsu 514-8507, Japan; t-yasuma0630@clin.medic.mie-u.ac.jp (T.Y.); dalessac@clin.medic.mie-u.ac.jp (C.N.D.-G.); 3 Department of Pulmonary and Critical Care Medicine, Faculty and Graduate School of Medicine, Mie University, Tsu 514-8507, Japan; kobayashitetsu@hotmail.com

**Keywords:** pembrolizumab, thymic carcinoma, squamous cell carcinoma, lung cancer

## Abstract

Thymic carcinoma is a rare malignant tumor with a poor prognosis. No standard treatment is currently available. The present case was a 64-year-old male smoker with no symptoms referred to our hospital because of abnormal chest radiological findings. The CT study showed a tumor between the anterior mediastinum and the right lung upper lobe, multiple nodular shadows along the right pleura, and pleural effusion. A CT-guided needle biopsy revealed squamous cell carcinoma. However, the differential diagnosis between thymic carcinoma and primary lung cancer was difficult. Treatment with carboplatin, nanoparticle albumin-bound paclitaxel, and pembrolizumab was initiated. The CT scan showed tumor shrinkage and good clinical response after four treatment cycles. Therapy was switched to maintenance therapy with pembrolizumab alone. Imaging studies showed further tumor shrinkage after twelve cycles of maintenance therapy with pembrolizumab. Sixteen cycles of maintenance therapy were continued without performance status deterioration. An abnormal radiological finding was detected after a twelve-month exacerbation-free period. The diagnosis was thymic carcinoma. Treatment with lenvatinib was initiated, and tumor-size reduction was observed. This is the first report of a case showing a successful maintenance therapy with pembrolizumab after effective first-line therapy with a combination of carboplatin-based chemotherapy plus pembrolizumab in advanced thymic carcinoma.

## 1. Introduction

Thymic carcinoma is a rare malignant tumor. The frequency of thymoma is 0.13 per 100,000 population [1]. Thymic carcinoma represents 15–20% of all thymomas [1]. Thymic carcinoma has a worse prognosis than thymoma. Based on the Masaoka classification, the 5-year overall survival is 100%, 81%, 51%, 24%, and 17% for stages I, II, III, IVa, and IVb, respectively [2]. There is a scarcity of evidence-based therapy because of the rarity of the disease. The therapy is generally similar to thymoma and includes a combination of cisplatin, doxorubicin, cyclophosphamide, vincristine, or carboplatin plus paclitaxel [3,4]. Systemic chemotherapy with carboplatin plus paclitaxel is less toxic than combination therapy with cisplatin, doxorubicin, cyclophosphamide, and vincristine. This combination therapy is recommended as the first-line therapy for thymic carcinoma by the National Comprehensive Cancer Network clinical practice guidelines in oncology [5]. In addition, the expression of PD-L1 is high in thymic carcinoma compared to thymoma, and this may explain the therapeutic efficacy of pembrolizumab, a PD-1 inhibitor, in this tumor [6,7]. The National Comprehensive Cancer Network clinical practice guidelines in oncology recommends pembrolizumab as a second-line treatment for thymic carcinoma. Squamous cell carcinoma is the most common histological type of thymic carcinoma, and therefore, differential diagnosis with primary squamous cell lung carcinoma is sometimes difficult [8]. In patients with primary squamous cell lung carcinoma and good performance status (performance status 0–1), the addition of immunotherapy to platinum-based chemotherapy (carboplatin, nanoparticle albumin-bound paclitaxel, pembrolizumab (Keynote-407)) improves progression-free survival and overall survival. Thus, this combination therapy is considered a standard therapeutic approach for this type of lung cancer [9]. The use of this standard therapy for lung cancer has not been reported for the treatment of thymic carcinoma. This report is the first case of thymic carcinoma treated with carboplatin, nanoparticle albumin-bound paclitaxel, and pembrolizumab as first-line therapy followed by a maintenance therapy with pembrolizumab. 

## 2. Case Presentation

**Figure 1:**
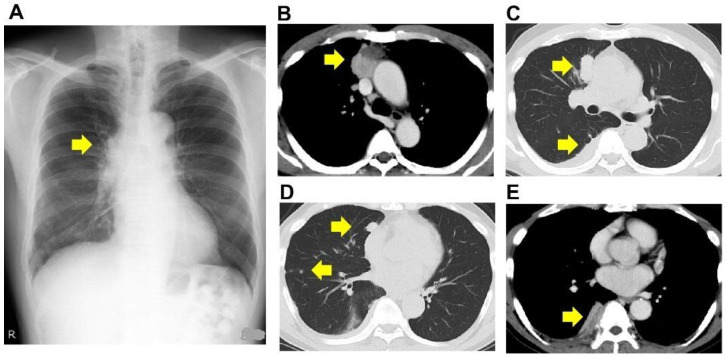
Radiological findings. Plain radiograph (**A**) and computed tomography (**B**–**E**). The computed tomography revealed a tumor between the anterior mediastinum and the right lung upper lobe (**B**,**C**), multiple nodular shadows along the right pleura (**D**), and pleural effusion (**E**). Arrows indicate the lesion.

The patient was a 64-year-old male and ex-smoker. The patient was a smoker (20 cigarettes per day) between the ages of 20 and 25. He was being treated for bronchial asthma with inhaled corticosteroids and long-acting beta-agonists. His Eastern Cooperative Oncology Group Performance Status score was 0. He was referred to our institution for an abnormal shadow on the chest X-ray taken during a routine medical check-up. He had no symptoms, and the routine laboratory examination showed normal findings. He had no occupational or environmental exposure to any noxious agent. The computed tomography (CT) study showed a tumor between the anterior mediastinum and the right lung upper lobe, multiple nodular shadows along the right pleura, and pleural effusion (Figure 1A–E).

**Figure 2:**
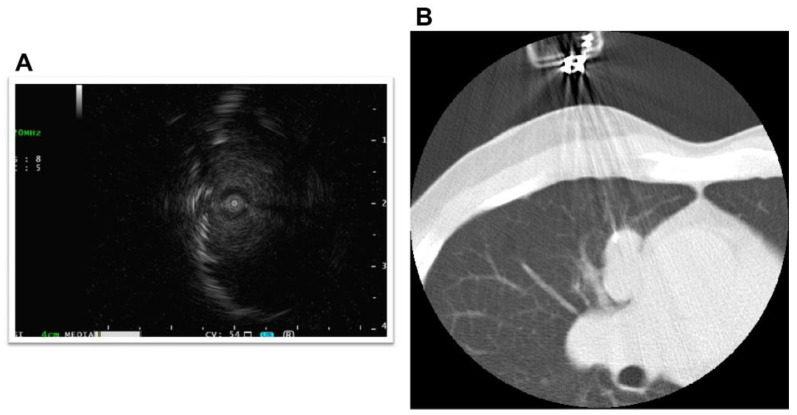
Bronchoscopy and computed tomography-guided biopsy. A thin bronchoscope, endobronchial ultrasonography, and a guide sheath were used during the bronchoscopy (**A**). Biopsy was performed under computed tomography guidance (**B**).

**Figure 3:**
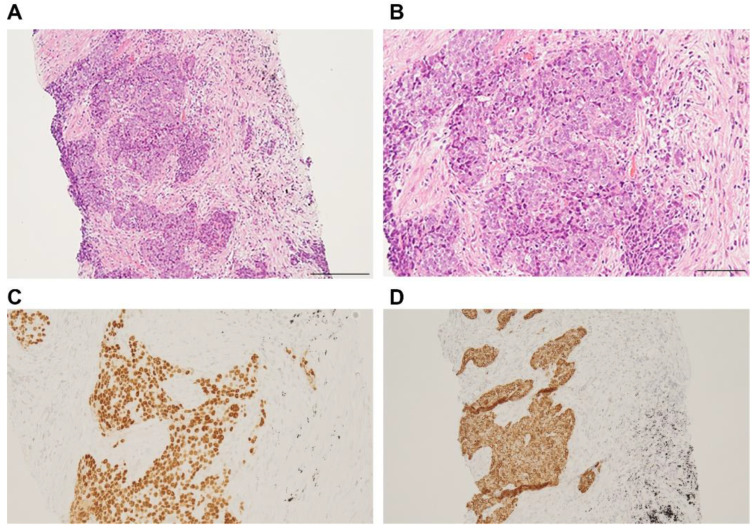
Tumor pathological findings. Hematoxylin/eosin staining of the tissue specimen revealed a solid tumor with interstitial fibrosis (**A**,**B**) and positive immunostaining for p40 (**C**) and CK5/6 (**D**). Scale bars indicate 200 µm (**A**) and 80 µm (**B**).

**Figure 4:**
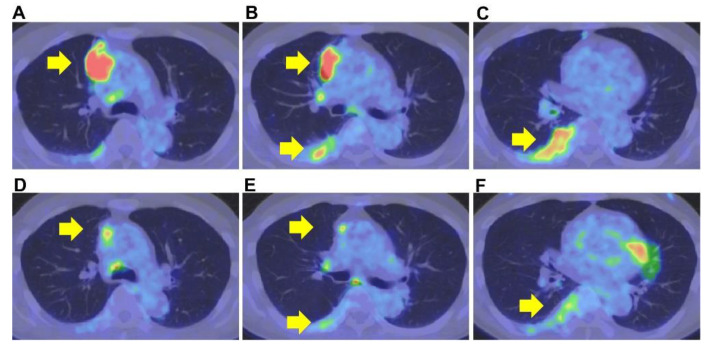
Positron emission tomography–computed tomography. Before therapy, the primary tumor’s positron emission tomography–computed tomography showed high ^18^F-fluorodeoxyglucose accumulation and a maximum standardized uptake value of 10.95 (**A**–**C**). After therapy, the primary tumor’s positron emission tomography–computed tomography showed a maximum standardized uptake value of 4.5 (**D**–**F**).

The primary lesion was located between the mediastinum and the right lung lobe, thus, it was difficult to distinguish whether the tumor originated from the mediastinum or the lungs. A thin bronchoscope with endobronchial ultrasonography and a guide sheath was inserted into the right B3biαx bronchial branch, and a biopsy within the tumor was performed. However, the histopathological study showed no diagnostic findings. CT-guided needle biopsy (CTNB) was then performed (Figure 2A,B). An expert pathologist revised the tissue specimen sampled by CTNB. The pathology report revealed a solid tumor with malignant squamous cells and scarring tissue and positive immunostaining for p40 and CK5/6. The pathological diagnosis was non-keratinized squamous cell carcinoma (Figure 3A–D). The differential diagnosis between thymic carcinoma and primary lung cancer was difficult based on imaging and histopathological findings. A PET/CT scan showed a primary tumor with high ^18^F-fluorodeoxyglucose accumulation, a maximum standardized uptake value of 10.95, and multiple nodules with a maximum standardized uptake value of 7.08 in the right lung interlobar region and pleura. The diagnosis was cancerous pleural dissemination (Figure 4A–F). The tumor was negative for epidermal growth factor receptor (*EGFR*) gene mutation, anaplastic lymphoma kinase (*ALK*) fusion gene, and the proto-oncogene tyrosine-protein kinase (*ROS1*). The degree of tumor PD-L1 (22C3) staining was low (5–10%). The tumor was considered unresectable. 

The present case was discussed by a multidisciplinary team that consisted of pulmonologists from our clinical department, radiologists, and thoracic surgeons to decide the most effective therapeutic approach for either type of tumor. If the tumor were lung cancer, the clinical stage would be cT4N0M1 Stage IVA based on the American Joint Committee on Cancer (AJCC) staging system version 8, and the first-line treatment would be the Keynote 407 regimen (carboplatin + nanoparticle albumin-bound paclitaxel + pembrolizumab) [10]. On the other hand, if the tumor were thymic carcinoma, the clinical stage would be cT4N0M1 Stage IVA based on the AJCC staging system version 8, and the first-line treatment would be carboplatin combined with paclitaxel [10]. In two clinical trials, pembrolizumab was effective against thymic carcinoma and is recommended as second-line treatment by the National Comprehensive Cancer Network Clinical Practice Guidelines in Oncology. Based on these considerations, treatment with carboplatin, nanoparticle albumin-bound paclitaxel, and pembrolizumab was started. After two weeks of follow-up, a CT scan showed a reduction in tumor size with no nausea, malaise, severe hematological toxicity, abnormal electrocardiographic findings, or immune-related complications. The patient was discharged. 

**Figure 5:**
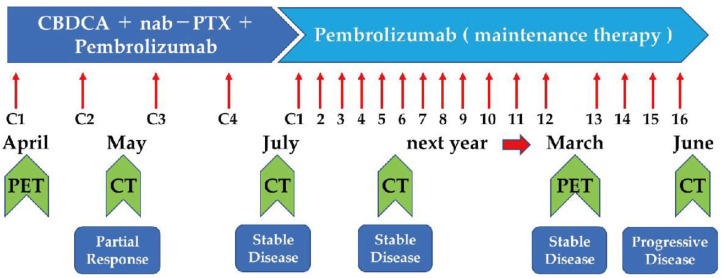
The clinical course of the patient. PET, positron emission tomography; CT, computed tomography; C, chemotherapy, CBDCA, carboplatin; PTX, paclitaxel; nab, nanoparticle albumin-bound.

CT scan showed tumor size reduction (partial response) after two cycles of combination therapy at the outpatient department, and a CT scan performed after four additional cycles of combination therapy showed no deterioration. Therefore, therapy was changed to maintenance therapy with pembrolizumab. The tumor size was gradually reduced following maintenance therapy with pembrolizumab (Figure 5). A PET/CT scan performed after twelve pembrolizumab cycles showed substantial tumor size reduction with decreased standardized uptake values (Figure 4). Maintenance therapy was continued for up to sixteen cycles due to a good response to the therapy and the patient’s stable performance status. Fourteen months after starting the therapy, the patient had a twelve-month exacerbation-free period. After that, the patient had an exacerbation of his disease that was treated with a combination of cisplatin, doxorubicin, cyclophosphamide, and vincristine as a second-line therapy and subsequently with amrubicin as third-line therapy. Due to the patient’s strong desire, pembrolizumab was re-administered as a fourth-line therapy, although no response was observed. Therapy with lenvatinib was then indicated, and the clinical response was good. Lenvatinib is an oral multi-target kinase inhibitor. The use of lenvatinib was associated with a good response rate (38%) in thymic carcinoma after platinum-based chemotherapy [11]. In the current case, lenvatinib was administered as fifth-line therapy without any PS impairment. Good tumor response is expected with the ongoing Phase 2 trials with pembrolizumab and lenvatinib for B3 thymoma or thymic carcinoma after platinum-based chemotherapy (PECATI study) [12]. In addition, platin-based chemotherapy combined with pembrolizumab (NCT04554524) as first-line therapy for patients with thymoma and thymic carcinoma is also currently underway (https://clinicaltrials.gov/ct2/show/NCT04554524 accessed on 7 July 2022). 

CT-guided biopsy of a pleural region showing high accumulation by PET-CT scan was performed. The same pathologist that revised the first tumor specimen evaluated the tissue sample. The pathology report described the presence of malignant squamous cells with areas of fibrotic tissues but without keratin. Next-generation sequencing (NGS) analysis was negative for gene abnormalities commonly observed in lung cancer, including *RET* fusion gene and *BRAF* mutation. The multidisciplinary team and the pathologist considered that based on the histopathological findings, the lack of proto-oncogene mutations, and the good response to lenvatinib, the present case is compatible with the diagnosis of thymic carcinoma. The patient is alive and has a more than 39 months survival time. This survival time is longer than previously reported cases of thymic carcinoma [13,14]. 

## 3. Discussion

Thymic tumors are rare and thymic carcinomas are even less common than thymomas [1]. Therefore, it is difficult to establish standard therapeutic guidelines for thymic tumors. Thymic carcinoma has more malignant behavior than thymomas. The combination therapy of carboplatin with paclitaxel is recommended for treating thymic carcinoma due to its efficacy and toxicity profile. Peripheral neuropathy is a common adverse event in patients receiving paclitaxel. However, a low frequency of side effects, including neutropenia and peripheral neuropathy, has been reported in phase III clinical trials with a combination of carboplatin and nanoparticle albumin-bound paclitaxel for advanced non-small-cell lung cancer [15]. Efficacy of carboplatin plus nanoparticle albumin-bound paclitaxel was also previously reported for advanced thymic carcinoma [16]. However, even though this therapy may be effective, the drugs cannot be administered long term to control the disease progression because they are cytotoxic. Thymic epithelial tumors have high PD-L1 expression. PD-L1 positive rates of 70% for thymic carcinoma and 23% for thymoma have been reported [7]. Therefore, the efficacy of PD-L1 inhibitors is predictable [7]. Giaccone et al. reported in a phase II trial that pembrolizumab monotherapy as a second-line therapy is effective in patients with thymic carcinoma [6]. They observed a response rate of 22.5%, a progression-free survival of 4.2 months, and prolonged median survival in patients having tumors with PD-L1 expression of 50% or more (median survival: 24 months vs. 2.9 months) compared to those with less PD-L1 expression [6]. Another study showed that immunotherapy is associated with a 5-year survival rate of 18% and long-lasting effects without increased complications in patients with thymic carcinoma [17]. 

Thomas et al. reported a progression-free survival of 7.9 months in patients with thymic carcinoma with a low PD-L1 expression treated with a combination of platinum-based chemotherapy (carboplatin plus pemetrexed) and pembrolizumab as a third-line therapy followed by maintenance therapy with pembrolizumab plus pemetrexed [18]. Thomas et al. believe that a chemoimmunotherapy combination is an effective therapeutic approach for thymic carcinoma with low PD-L1 expression [18]. In the current case, whether the tumor was thymic carcinoma or lung cancer was difficult to distinguish because the histological type of the tumor was squamous cell carcinoma, and the tumor was located between the mediastinum and the lung field. Immunostaining of Kit and CD5 has been recommended to differentiate thymic carcinoma from lung cancer. The immunoreactivity of Kit and CD5 is 80% and 70% in thymic carcinoma and 20% and 15% in lung cancer, respectively [19]. However, we did not perform immunostaining of these factors because of the reported high rate of false-positive results. Although the differential diagnosis between thymic carcinoma and lung cancer was unclear, we needed to define the therapeutic strategy. The Keynote-407 trial has demonstrated the efficacy of the combined therapy of platinum-based chemotherapy (carboplatin + nanoparticle albumin-bound paclitaxel, or carboplatin + paclitaxel) plus pembrolizumab as first-line therapy for lung squamous cell carcinoma [9,20]. Although the response of lung cancer to pembrolizumab monotherapy is not as good when the PD-L1 expression is low as when the PD-L1 expression is high, even with no high PD-L1 expression, significant prolongation of progression-free survival and overall survival is achieved with the combination of pembrolizumab and platinum-based chemotherapy followed by maintenance therapy with pembrolizumab [20]. In the current case, despite the low expression of PD-L1, the tumor continued shrinking in size without any immune-related complications even after changing to pembrolizumab monotherapy alone. Three-phase II clinical trials with PD-1 inhibitors have been reported in patients with thymic carcinoma [6,21]. The response rate was between 19.2 and 22.5% [7,21] with pembrolizumab but 0% with nivolumab [6,21,22]. The reason for this discrepancy is unclear, but differences in ethnicity, treatment history, or tumor histological type may be the potential explanations. Maintenance of performance status is an outstanding advantage of immunotherapy. However, serious complications such as myocarditis have been reported with immunotherapy [6,17]. Therefore, caution and careful monitoring is required during immunotherapy [6,17]. 

## 4. Conclusions

This report is the first to show a successful maintenance therapy with pembrolizumab after effective first-line therapy with a combination of carboplatin-based chemotherapy plus pembrolizumab in advanced thymic carcinoma.

## Data Availability

All data are available upon reasonable request to the corresponding author.
